# Supplementary material for: Immune-privileged tissues formed from immunologically cloaked mouse embryonic stem cells survive long term in allogeneic hosts
Source: Nat Biomed Eng. 2023 Nov 23;8(4):427–42. doi: 10.1038/s41551-023-01133-y (PMC11087263; doi:10.1038/s41551-023-01133-y)
Supplement: Supplementary file 1 — Supplementary tables, figure and methods. [file 41551_2023_1133_MOESM1_ESM.pdf]

nature biomedical engineering

Article

https://doi.org/10.1038/s41551-023-01133-y

# Immune-privileged tissues formed from immunologically cloaked mouse embryonic stem cells survive long term in allogeneic hosts

In the format provided by the authors and unedited

**Supplementary Table 1 I** Mouse and human immunomodulatory transgenes with NCBI RefSeq protein ID.

| Gene CDS | species | RefSeq protein ID |
|---|---|---|
| *mPdl1* | mouse | NP_054862.1 |
| *mCd200* | mouse | NP_034948.3 |
| *mCd47* | mouse | NP_001768.1 |
| *mH2-M3* | mouse | NP_038847.1 |
| *mFasl* | mouse | NP_034307.1 |
| *mMfge8* | mouse | NP_032620.2 |
| *mSerpinb9* | mouse | NP_033282.1 |
| *mCcl21b* | mouse | NP_001365534.1 |
| *hPDL1* | human | NP_054862.1 |
| *hCD200* | human | NP_001004196.2 |
| *hCD47* | human | NP_001768.1 |
| *hHLA-G1* | human | NP_001350496.1 |
| *hFASLG* | human | NP_000630.1 |
| *hMFGE8* | human | NP_005919.2 |
| *hSERPINB9* | human | NP_004146.1 |
| *hCCL21* | human | NP_002980.1 |

**Supplementary Table 2 l** Forward and reverse qPCR primers to detect expression of inserted immunomodulatory genes, differentiated cell markers, and housekeeper control genes.

| Gene CDS | qPCR Primer F | qPCR primer R |
|---|---|---|
| *mPdl1* | GCGAATCACGCTGAAAGTCAATGC | ACGGGTTGGTGGTCACTGTTTG |
| *mCd200* | CTCTCCACCTACAGCCTGATT | AGAACATCGTAAGGATGCAGTTG |
| *mCd47* | TGCGGTTCAGCTCAACTACTG | GCTTTGCGCCTCCACATTAC |
| *mH2-M3* | ACTATCAGGCTGCGTATGATGG | ACTCTAAACGGCTCTTCGTGA |
| *mFasl* | TGGTTGGAATGGGATTAGGA | GTGGGGGTTCCCTGTTAAAT |
| *mMfge8* | CCTGGGCCTGAAGAATAACA | GCATTGATCTTGCCCTGATT |
| *mSerpinb9* | TAGAGTGGCCAACAGGCTCT | GGAGCCACCTGACAACAACT |
| *mCcl21* | GTGATGGAGGGGGTCAGGA | GGGATGGGACAGCCTAAACT |
| *mEef2* | GTATTAAGAGCTGCGACCCC | ATAGAAGCGGCCTTTGTCAG |
| *hPDL1* | TGGCATTTGCTGAACGCATTT | TGCAGCCAGGTCTATTGTTTT |
| *hCD200* | GCTCTCTGCAAAATGCCCAG | CCATGGTTCTCGCTGAAGGT |
| *hCD47* | TCCGGTGGTATGGATGAGAAA | ACCAAGGCCAGTAGCATTCTT |
| *hHLA-G* | CACGCACAGACTGACAGAATG | GTCGCAGCCAATCATCCACT |
| *hFASL* | CCTTGGTAGGATTGGGCCTG | CTGGCTGGTAGACTCTCGGA |
| *hMFGE8* | CCTGCCACAACGGTGGTTTAT | CACATTTCGTCTCACAGTGGTT |
| *hSERPINB9* | AAGTGGAATGAACCGTTTGACG | GTGGCCTCCTGATACATCATCT |
| *hCCL21* | TCTCAGGCAGAGCTATGTGC | CTGTGACCGCTCAGTCCTCTT |
| *hOCT4* | CCCTCGTGCAGGCCCGAAAG | CCCAAGCTGCTGGGCGATGT |
| *hNANOG* | CCTTGGCTGCCGTCTCTGGC | AGCAAAGCCTCCCAATCCCAAACA |
| *hSOX17* | GTGGACCGCACGGAATTTG | GGAGATTCACACCGGAGTCA |
| *hFOXA2* | GGAGCAGCTACTATGCAGAGC | CGTGTTCATGCCGTTCATCC |
| *hYWAHZ* | CCGCCAGGACAAACCAGTAT | ACTTTTGGTACATTGTGGCTTCAA |
| *hGADPhH* | CATGAGAAGTATGACAACAGCCT | AGTCCTTCCACGATACCAAAGT |

**Supplementary Table 3 l** Tabulation of all experiments in which Klg-1 and Klg-2 mESCs were transplanted into isogeneic and allogeneic recipients. Table shows number of recipients injected, number of recipients that formed teratomas, and the length of observation before euthanasia for each cage among all transplantation. Data corresponds to main Fig. 3c-e and Supplementary Fig. 2.

| **Donor → Recipient strain condition** | **Cage ID** | **# mice injected** | **# mice formed teratomas** | **# months before sacked** |
|---|---|---|---|---|
| **uncloaked FS B6N mESCs → BN, isogeneic** | C7003871 | 2 | 1 | 2 months |
| " " | C7003902 | 2 | 2 | 2 months |
| " " | C7003790 | 5 | 5 | 3 months |
| " " | C7170476 | 5 | 5 | 3 months |
| " " | C7212976 | 5 | 4 | 3 months |
| " " | C7283968 | 3 | 3 | 3 months |
| " " | C7658219 | 5 | 5 | 6 months |
| " " | C7658226 | 5 | 4 | 6 months |
| " " | C7720221 | 5 | 5 | 2 months |
| " " | C7658242 | 5 | 5 | 2 months |
| " " | C7912243 | 3 | 3 | 6 months |
| " " | C7912258 | 3 | 3 | 6 months |
| " " | C8200160 | 5 | 2 | 4 months |
| " " | C8200187 | 4 | 3 | 4 months |
| " " | C8261223 | 5 | 3 | 4 months |
| " " | C8261206 | 5 | 5 | 4 months |
| " " | C8321799 | 5 | 3 | 5 months |
| " " | C8321813 | 5 | 4 | 3 months |
| **Klg-1 mESCs → B6N, isogeneic** | C7003800 | 5 | 5 | 5 months |
| " " | C7003788 | 5 | 5 | 5 months |
| " " | C7003939 | 3 | 3 | 2 months |
| " " | C7003863 | 5 | 5 | 6 months |
| " " | C7212969 | 5 | 5 | 5 months |
| " " | C7212982 | 5 | 5 | 6 months |
| " " | C7283975 | 5 | 5 | 4 months |
| " " | C7283981 | 5 | 5 | 4 months |
| " " | C7283952 | 5 | 5 | 5 months |
| " " | C7420625 | 3 | 3 | 5 months |
| " " | C7524032 | 5 | 5 | 6 months |
| " " | C7524050 | 5 | 5 | 6 months |
| " " | C7720213 | 5 | 5 | 2 months |
| " " | C7658235 | 5 | 5 | 2 months |
| " " | C7749067 | 5 | 5 | 2 months |
| " " | C8200194 | 4 | 4 | 4 months |
| " " | C8185883 | 5 | 4 | 4 months |
| " " | C8261186 | 5 | 5 | 4 months |
| " " | C8261247 | 5 | 4 | 5 months |
| " " | C8321752 | 5 | 5 | 2 months |
| " " | C8321809 | 5 | 5 | 2 months |
| **Klg-1 mESCs → FVB, allogeneic** | C7003676 | 5 | 5 | 2 months |
| " " | C7003703 | 5 | 5 | 9 months |
| " " | C7003742 | 4 | 3 | 4 months |
| " " | C7143911 | 4 | 4 | 7 months |
| " " | C7238930 | 4 | 4 | 6 months |
| " " | C7308893 | 2 | 1 | 2 months |
| " " | C7308903 | 3 | 0 | n/a |
| " " | C7698704 | 5 | 5 | 5 months |
| " " | C7924439 | 5 | 4 | 3 months |
| " " | C7924456 | 5 | 2 | 4 months |
| " " | C7667371 | 3 | 3 | 6 months |

| | | | | |
|---|---|---|---|---|
| “ “ | C7667385 | 5 | 3 | 7 months |
| “ “ | C7766817 | 3 | 2 | 4 months |
| “ “ | C8268940 | 5 | 2 | 3 months |
| “ “ | C8268938 | 5 | 4 | 2 months |
| “ “ | C8334503 | 5 | 1 | 2 months |
| “ “ | C8334542 | 5 | 5 | 3 months |
| **Klg-1 mESCs → C3H, allogeneic** | C7434570 | 5 | 3 | 4 months |
| “ “ | C7744419 | 3 | 3 | 2 months |
| “ “ | C7685455 | 4 | 2 | 4 months |
| “ “ | C7744403 | 3 | 2 | 4 months |
| “ “ | C7685440 | 2 | 2 | 5 months |
| “ “ | C7633765 | 5 | 1 | 4 months |
| “ “ | C7633754 | 4 | 4 | 4 months |
| “ “ | C8269025 | 4 | 3 | 3 months |
| “ “ | C8269039 | 5 | 5 | 3 months |
| “ “ | C8213761 | 5 | 3 | 3 months |
| “ “ | C8334463 | 5 | 5 | 3 months |
| “ “ | C8334471 | 5 | 5 | 4 months |
| **Klg-1 mESCs → Balb/C, allogeneic** | C8542655 | 5 | 4 | 4 months |
| “ “ | C8542638 | 5 | 4 | 4 months |
| “ “ | C8542640 | 5 | 0 | no teratomas |
| “ “ | C8542629 | 5 | 4 | 4 months |
| **Klg-1 mESCs → CD1 allogeneic** | C7170495 | 5 | 3 | 7 months |
| “ “ | C7170482 | 5 | 0 | n/a |
| “ “ | C7283934 | 4 | 3 | 5 months |
| “ “ | C7283947 | 5 | 1 | 6 months |
| “ “ | C7215283 | 5 | 2 | 8 months |
| “ “ | C7283923 | 3 | 0 | no teratomas |
| “ “ | C7436427 | 5 | 0 | no teratomas |
| **Klg-2 mESCs → C57BL/6N, isogeneic** | C8185877 | 5 | 5 | 3 months |
| “ “ | C8185865 | 5 | 5 | 3 months |
| “ “ | C7524045 | 5 | 5 | 6 months |
| “ “ | C8261210 | 5 | 5 | 3 months |
| “ “ | C8261193 | 5 | 5 | 3 months |
| “ “ | C8321781 | 5 | 3 | 3 months |
| “ “ | C8321775 | 5 | 3 | 3 months |
| **Klg-2 mESCs → FVB, allogeneic** | C7459341 | 2 | 1 | 3 months |
| “ “ | C7496163 | 5 | 3 | 6 months |
| “ “ | C7443801 | 5 | 0 | no teratomas |
| “ “ | C8116956 | 5 | 0 | no teratomas |
| “ “ | C8268964 | 5 | 0 | no teratomas |
| “ “ | C8268972 | 5 | 0 | no teratomas |
| “ “ | C8334492 | 5 | 1 | 3 months |
| “ “ | C8334519 | 5 | 0 | no teratomas |
| **Klg-2 mESCs → C3H, allogeneic** | C7538143 | 5 | 3 | 4 months |
| “ “ | C7538136 | 5 | 3 | 4 months |
| “ “ | C7434558 | 5 | 2 | 5 months |
| “ “ | C8135726 | 2 | 2 | 6 months |
| “ “ | C7967151 | 2 | 2 | 4 months |
| “ “ | C7848218 | 2 | 2 | 6 months |

| | | | | |
|---|---|---|---|---|
| “ “ | C8213816 | 5 | 2 | 5 months |
| “ “ | C8213828 | 5 | 2 | 5 months |
| “ “ | C8213837 | 5 | 4 | 5 months |
| “ “ | C8268986 | 4 | 2 | 4 months |
| “ “ | C8269002 | 5 | 0 | no teratomas |
| “ “ | C8334485 | 5 | 4 | 3 months |
| “ “ | C8334437 | 5 | 1 | 4 months |

**Supplementary Table 4 l** Number and position of piggyBac insertions, each containing an immunomodulatory transgene, in Klg-1mESCs. Data was generated using splinkerette PCR with primers against the long terminal repeats of piggyBac flanking and comparing the next generation (NGS)- sequenced amplified products to a mouse reference genome. Complete description in methods. Table classes defined by number of reads on each TTAA-flanking side. All classes have greater than 10 reads on one side; class a, one read on one other side; class b, 2-4 reads on other one side; class c, more than 4 reads on other side one side.

| # | Chr | Start position | End position | associated gene (basal + extension) | associated gene (1kb upstream, 10kb down | class |
|---|---|---|---|---|---|---|
| 1 | chr1 | 191717633 | 191717637 | Lpgat1 (-754) | Lpgat1 (-754) | a |
| 2 | chr1 | 134405686 | 134405690 | Cyb5r1 (-93) | Cyb5r1 (-93) | a |
| 3 | chr1 | 84829501 | 84829505 | Dner (-133282), Trip12 (+9801) | Trip12 (+9801) | a |
| 4 | chr1 | 23153142 | 23153146 | 4933415F23Rik (-50891), Gm6420 (+103262) | NONE | a |
| 5 | chr2 | 180071074 | 180071078 | Gtpbp5 (-717) | Gtpbp5 (-717) | a |
| 6 | chr5 | 107123013 | 107123017 | Cdc7 (+158454), Tgfbr3 (+166614) | NONE | a |
| 7 | chr5 | 73011177 | 73011181 | Slc10a4 (+4275), Fryl (+245409) | Slc10a4 (+4275) | a |
| 8 | chr6 | 32083096 | 32083100 | 1700012A03Rik (+32852), Plxna4 (+505094) | NONE | a |
| 9 | chr6 | 16840125 | 16840129 | Tfec (+58314) | NONE | a |
| 10 | chr7 | 138527843 | 138527847 | Tcerg1l (-130117), Mapk1ip1 (+318428) | NONE | a |
| 11 | chr7 | 114902623 | 114902627 | Gm6816 (+25101), Insc (+156859) | NONE | a |
| 12 | chr7 | 36768266 | 36768270 | Tshz3 (+70150) | NONE | a |
| 13 | chr8 | 124648197 | 124648201 | 2310022B05Rik (+15170), Capn9 (+72088) | NONE | a |
| 14 | chr10 | 113185466 | 113185470 | Atxn7l3b (-256442) | NONE | a |
| 15 | chr10 | 67187286 | 67187290 | Jmjd1c (+60030), Nrbf2 (+98017) | NONE | a |
| 16 | chr11 | 72859864 | 72859868 | Atp2a3 (-101303), Zzef1 (+63612) | NONE | a |
| 17 | chr12 | 72744695 | 72744699 | Dhrs7 (-79869), Ppm1a (-16514) | NONE | a |
| 18 | chr14 | 76625222 | 76625226 | Serp2 (-68537), Lacc1 (+411417) | NONE | a |
| 19 | chr14 | 70749859 | 70749863 | Npm2 (-96777), Xpo7 (+16767) | NONE | a |
| 20 | chr15 | 74654046 | 74654050 | Mroh4 (-17787), Arc (+18520) | NONE | a |
| 21 | chr16 | 20360201 | 20360205 | Cyp2ab1 (-37138), Abcc5 (+66191) | NONE | a |
| 22 | chr17 | 33355492 | 33355496 | Zfp81 (+3384), Zfp955b (+65845) | Zfp81 (+3384) | a |
| 23 | chr18 | 23803174 | 23803178 | Mapre2 (-808) | Mapre2 (-808) | a |
| 24 | chr19 | 55896945 | 55896949 | Habp2 (-390975), Tcf7l2 (+155137) | NONE | a |
| 25 | chr19 | 5327933 | 5327937 | Catsper1 (-7806), Gal3st3 (+29604) | NONE | a |
| 26 | chr1 | 161781388 | 161781392 | Fasl (+7105), Tnfsf18 (+286735) | Fasl (+7105) | c |
| 27 | chr1 | 132237685 | 132237689 | Klhdc8a (-60939), Gm10188 (-7920) | NONE | c |
| 28 | chr2 | 50914093 | 50914097 | Mmadhc (-617378), Rnd3 (+235016) | NONE | c |
| 29 | chr3 | 98013125 | 98013129 | Notch2 (-411) | Notch2 (-411) | c |
| 30 | chr4 | 41129612 | 41129616 | Ube2r2 (-6129), Nol6 (-5159) | NONE | c |
| 31 | chr8 | 117017967 | 117017971 | Gcsh (-24432), Pkd1l2 (+64480) | NONE | c |
| 32 | chr10 | 38610142 | 38610146 | Rfpl4b (+211635) | NONE | c |
| 33 | chr11 | 120598854 | 120598858 | Alyref (-491), Anapc11 (+435) | Alyref (-491), Anapc11(+435) | c |
| 34 | chr11 | 88966965 | 88966969 | Scpep1 (-11502), Coil (-6968) | NONE | c |
| 35 | chr11 | 31392342 | 31392346 | Stc2 (-22270), Bod1 (+279541) | NONE | c |
| 36 | chr11 | 15569072 | 15569076 | Vstm2a (-688650) | NONE | c |
| 37 | chr12 | 82470236 | 82470240 | Gm5435 (+26299), Sipa1l1 (+158889) | NONE | c |
| 38 | chr12 | 69177378 | 69177382 | Rpl36al (+6633), Lrr1 (+8566) | Rpl36al (+6633), Lrr1 (+8566) | c |

| | | | | | | |
|---|---|---|---|---|---|---|
| 39 | chr18 | 40060860 | 40060864 | Yipf5 (+158537), Pabpc2 (+287365) | NONE | c |
| 40 | chrX | 87579696 | 87579700 | Il1rapl1 (+535947) | NONE | c |
| 41 | chr1 | 128441620 | 128441624 | Dars (-24206), Cxcr4 (+150668) | NONE | b |
| 42 | chr1 | 86237174 | 86237178 | B3gnt7 (-66045), Armc9 (+82366) | NONE | b |
| 43 | chr1 | 47274114 | 47274118 | Slc39a10 (-420070) | NONE | b |
| 44 | chr2 | 129419766 | 129419770 | Il1b (-48629), F830045P16Rik (+116834) | NONE | b |
| 45 | chr2 | 129178651 | 129178655 | Slc20a1 (-20111), Chchd5 (+48953) | NONE | b |
| 46 | chr2 | 17067679 | 17067683 | Nebl (+663362), Plxdc2 (+711377) | NONE | b |
| 47 | chr3 | 64374755 | 64374759 | Vmn2r3 (-87340), Vmn2r4 (+35300) | NONE | b |
| 48 | chr3 | 30939259 | 30939263 | Phc3 (+30140), Gpr160 (+83311) | NONE | b |
| 49 | chr5 | 115001292 | 115001296 | Hnf1a (-30227), Sppl3 (-9843) | NONE | b |
| 50 | chr7 | 92870412 | 92870416 | Prcp (-4839), 4632434I11Rik (+3799) | 4632434I11Rik (+3799) | b |
| 51 | chr13 | 45691011 | 45691015 | Gmpr (+183569), Atxn1 (+273978) | NONE | b |
| 52 | chr14 | 14308741 | 14308745 | Olfr720 (-132626), Olfr31 (-19370) | NONE | b |
| 53 | chr15 | 35305533 | 35305537 | Vps13b (-66011), Osr2 (+9437) | Osr2 (+9437) | b |
| 54 | chr18 | 67161288 | 67161292 | Gnal (+72954), Mppe1 (+84540) | NONE | b |
| 55 | chr19 | 38967824 | 38967828 | Cyp2c55 (-39193), Hells (+36911) | NONE | b |
| 56 | chrX | 65503194 | 65503198 | Ctag2 (+455552), 4930447F04Rik (+801028) | NONE | b |

**Supplementary Table 5 |** List of antibodies and their respective ion tags used in CyTOF mass spectrometry to phenotype hPBMCs after co-culture with uncloaked FS or cloaked RPEs.

| Cytof Tag | Antibody Specificity. |
|---|---|
| 89 Y | CD45 |
| 141 Pr | HLA-DR |
| 142 Nd | cCaspase3 |
| 143 Nd | IgD |
| 144 Nd | CD27 |
| 145 Nd | CD11c |
| 146 Nd | CD8a |
| 147 Sm | CD20 |
| 148 Nd | CD4 |
| 151 Eu | CD123 |
| 152 Sm | CD3 |
| 153 Eu | CD45RA |
| 154 Sm | CD19 |
| 155 Gd | CD161 |
| 156 Gd | CD38 |
| 158 Gd | IL10 |
| 160 Gd | CD14 |
| 195 Pt | Cisplatin |
| 161 Dy | CD25 |
| 163 Dy | CD127 |
| 164 Dy | CD24 |
| 165 Ho | CD16 |
| 166 Er | CXCR5 |
| 167 Er | IL2 |
| 168 Er | IFNg |
| 169 Tm | CD66b |
| 170 Er | TNFa |
| 171 Yb | GZMB |
| 172 Yb | TCRgd |
| 173 Yb | CD56 |
| 174 Yb | CD45RO |
| 176 Yb | CCR7 |
| 191 Ir | DNA1 |
| 193 Ir | DNA2 |
| 194 Pt | Cisplatin |

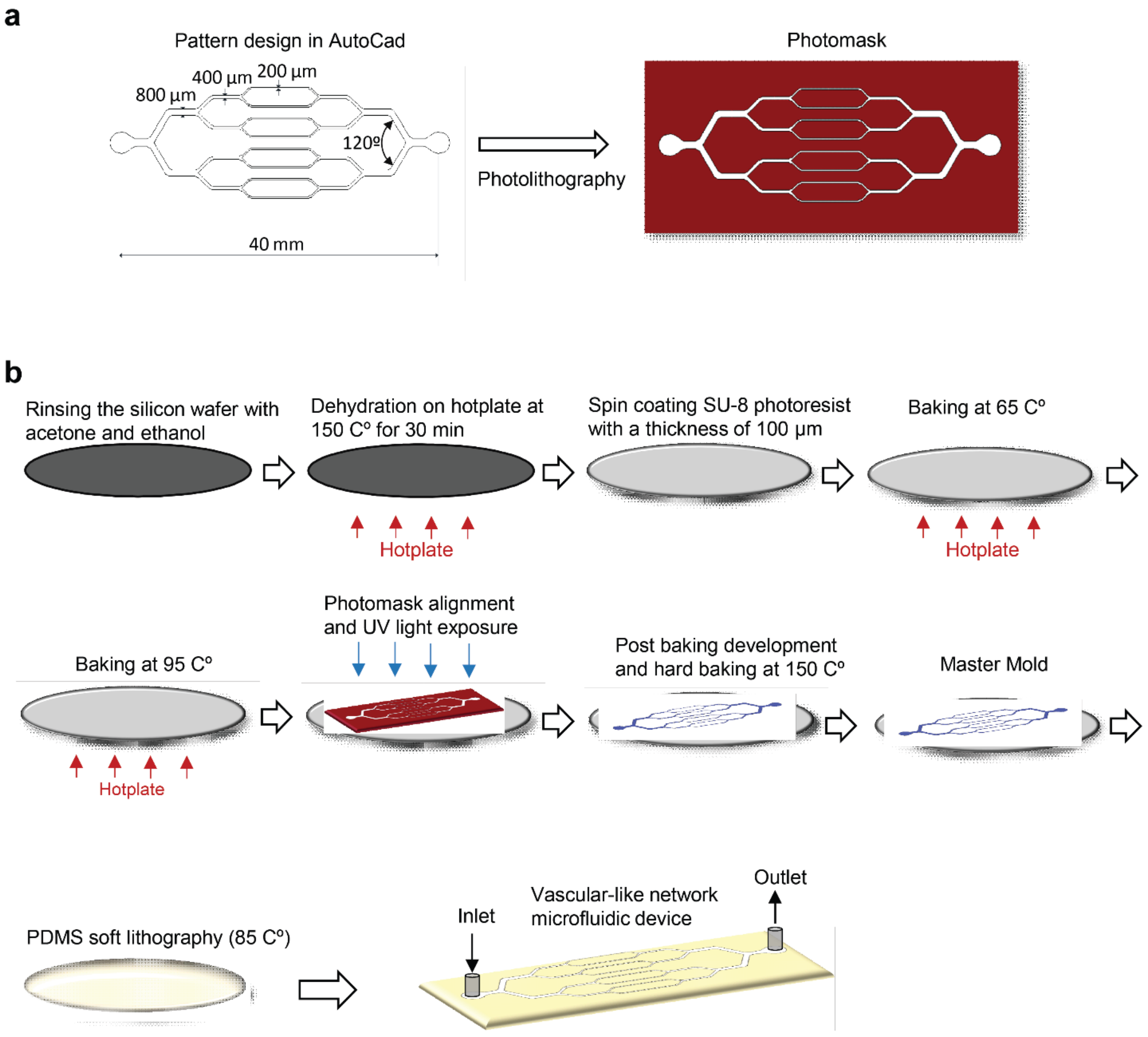


**Supplementary Figure 1. l Schematic design and fabrication procedure for the microfluidic device. a.** schematic diagram showing the AutoCad design with specifications of the pattern based on which the photomask is created. **b.** Photolithography and PDMS soft lithography procedures for the fabrication of the PDMS-based vascular-like network microfluidic device.

**Supplementary Methods**

**Manufacture of the Microfluidic device for loading Endothelial cells.** A microfluidic network consisting of a three-level branching microchannels was designed in AutoCad 2019 where the angles at the bifurcations was 120° and the width of the microchannels was 200, 400 and 800 µm, respectively (**Supplementary Fig. 1a**). The photomask was developed using standard photolithography. The device was fabricated using polydimethylsiloxane (PDMS) soft lithography as shown in **Supplementary Fig. 1b**. Briefly, a silicon wafer was rinsed with acetone and methanol followed by drying the wafer on a hot plate at 150°C over 30 min. SU-8 photoresist (SU8-3000, Microchem, Westborough, MA) was spun-coated over the silicon wafer with a thickness of 100 µm followed by a two-step baking on the hotplate at 65°C and 95°C, respectively. Then, the branching microchannel pattern was transferred to the SU-8 thin film by aligning the photomask and UV light exposure followed by post-baking, development, and hard baking at 150°C, which led to the master mold. PDMS (Slygard 184, Dow Chemical, Midland, MI) was prepared at the weight ratio of 10:1 under the vacuum for 1 hour. The PDMS was cast into the master mold to replicate the branching microchannel network followed by curing in an oven at 80°C for 2 hours. The inlet and outlet of the microfluidic device, which were used for cell loading, tube connection and culture media circulation, were made using a 1mm puncher.
