# Supplementary material for: Immune-privileged tissues formed from immunologically cloaked mouse embryonic stem cells survive long term in allogeneic hosts
Source: Nat Biomed Eng. 2023 Nov 23;8(4):427–42. doi: 10.1038/s41551-023-01133-y (PMC11087263; doi:10.1038/s41551-023-01133-y)
Supplement: Supplementary file 2 — Reporting Summary [file 41551_2023_1133_MOESM2_ESM.pdf]

nature portfolio

Corresponding author(s): Andras Nagy

Last updated by author(s): Sep 12, 2023

Reporting Summary

Nature Portfolio wishes to improve the reproducibility of the work that we publish. This form provides structure for consistency and transparency in reporting. For further information on Nature Portfolio policies, see our Editorial Policies and the Editorial Policy Checklist.

Statistics

For all statistical analyses, confirm that the following items are present in the figure legend, table legend, main text, or Methods section.

n/a	Confirmed	
☐	☒	The exact sample size (*n*) for each experimental group/condition, given as a discrete number and unit of measurement
☐	☒	A statement on whether measurements were taken from distinct samples or whether the same sample was measured repeatedly
☐	☒	The statistical test(s) used AND whether they are one- or two-sided<br>*Only common tests should be described solely by name; describe more complex techniques in the Methods section.*
☐	☒	A description of all covariates tested
☒	☐	A description of any assumptions or corrections, such as tests of normality and adjustment for multiple comparisons
☒	☐	A full description of the statistical parameters including central tendency (e.g. means) or other basic estimates (e.g. regression coefficient) AND variation (e.g. standard deviation) or associated estimates of uncertainty (e.g. confidence intervals)
☐	☒	For null hypothesis testing, the test statistic (e.g. *F*, *t*, *r*) with confidence intervals, effect sizes, degrees of freedom and *P* value noted<br>*Give P values as exact values whenever suitable.*
☒	☐	For Bayesian analysis, information on the choice of priors and Markov chain Monte Carlo settings
☒	☐	For hierarchical and complex designs, identification of the appropriate level for tests and full reporting of outcomes
☒	☐	Estimates of effect sizes (e.g. Cohen's *d*, Pearson's *r*), indicating how they were calculated

Our web collection on statistics for biologists contains articles on many of the points above.

Software and code

Policy information about availability of computer code

Data collection	Zen 2011 (black edition), for the confocal images taken for immunostaining<br>OpenLab 5.5.2, for bright-field images<br>Bio-Rad CFX Manager 3.1, for qPCR data collection<br>PerkenELmer Living Image V4.5, for Bioluminescent Imaging (BLI) collection<br>Summit v6.2, for FACS (Beckman Coulter, MoFlow AStrios EQ cell sorter);<br>Gallios software, for flow cytometry (Beckman Coulter, GALLIOS flow cytometer)
Data analysis	Bio-Rad CFX Manager 3.1, for qPCR analysis<br>GraphPad Prism software v10, for all data processing.<br>Kaluza 1.5 for and FlowJo v10, for flow-cytometry analysis<br>Cytobank software, for Cytof data analysis<br>PerkenELmer Living Image V4.5, for bioluminescence imaging (BLI) analysis<br>Star tool, for Splinkerette PCR analysis<br>Integrative Genomic Viewer (IGV), for Splinkeretee PCR analysis<br>Timmomatic for total RNA Seq, and Splinkerette for PCR analysis<br>FASQC, for total RNA Seq (https://www.bioinformatics.babraham.ac.uk/projects/fastqc)<br>Kalliso, for total RNA Seq analysis<br>DESeq2, for total RNA Seq analysis<br>R, for total RNA seq (http://www.r-project.org)

For manuscripts utilizing custom algorithms or software that are central to the research but not yet described in published literature, software must be made available to editors and reviewers. We strongly encourage code deposition in a community repository (e.g. GitHub). See the Nature Portfolio guidelines for submitting code & software for further information.

Data

Policy information about availability of data

All manuscripts must include a data availability statement. This statement should provide the following information, where applicable:

- Accession codes, unique identifiers, or web links for publicly available datasets
- A description of any restrictions on data availability
- For clinical datasets or third party data, please ensure that the statement adheres to our policy

The data for the teratoma-growth experiments (Figs. 2 and 4, and Extended Data Fig. 9) and for the blood-glucose experiments (Extended Data Fig. 10) are provided as Source Data. The raw and analysed datasets generated during the study are available for research purposes from the corresponding author on reasonable request.

Research involving human participants, their data, or biological material

Policy information about studies with human participants or human data. See also policy information about sex, gender (identity/presentation), and sexual orientation and race, ethnicity and racism.

Reporting on sex and gender	The study did not involve human participants.
Reporting on race, ethnicity, or other socially relevant groupings	—
Population characteristics	—
Recruitment	—
Ethics oversight	—

Note that full information on the approval of the study protocol must also be provided in the manuscript.

Field-specific reporting

Please select the one below that is the best fit for your research. If you are not sure, read the appropriate sections before making your selection.

☒ Life sciences ☐ Behavioural & social sciences ☐ Ecological, evolutionary & environmental sciences

For a reference copy of the document with all sections, see nature.com/documents/nr-reporting-summary-flat.pdf

Life sciences study design

All studies must disclose on these points even when the disclosure is negative.

Sample size	The number of samples used in different experiments was determined in accordance with similar studies in the field.
Data exclusions	No data were intentionally excluded.
Replication	Replication numbers and attempts were carried out as deemed necessary, and are reported in the paper.
Randomization	Samples were randomized when possible.
Blinding	Blinding was either not deemed necessary, owing to the detection method, or not possible owing to the nature of the experiment (such as growth of visible subcutaneous teratomas).

Reporting for specific materials, systems and methods

We require information from authors about some types of materials, experimental systems and methods used in many studies. Here, indicate whether each material, system or method listed is relevant to your study. If you are not sure if a list item applies to your research, read the appropriate section before selecting a response.

Materials & experimental systems

n/a	Involved in the study	
☐	☒	Antibodies
☐	☒	Eukaryotic cell lines
☒	☐	Palaeontology and archaeology
☐	☒	Animals and other organisms
☒	☐	Clinical data
☒	☐	Dual use research of concern
☒	☐	Plants

Methods

n/a	Involved in the study	
☒	☐	ChIP-seq
☐	☒	Flow cytometry
☒	☐	MRI-based neuroimaging

Antibodies

Antibodies used

Anti-mouse
anti-mouse CD3ε (BD Biosciences 553058)
anti-mouse CD28 (BD Biosciences 553295)
anti-mouse PD-L1 (alexa488, Novus NBP1-43262AF488)
anti-mouse CD47, (BV421, BD Biosciences 740055)
anti-mouse CD200 (alexa647, BD Biosciences 565544)
anti-mouse FASL (Novus NBP1-97519)
anti-mouse H2-M3 (BD Biosciences 551769)
anti-mouse SERPINB9 (HycultBiotech HP8035)
anti-mouse CCL21 (R&D AF457)
anti-mouse MFGE8 (Biolegend 518603)
anti-mouse CD31 (Biolegend 102417)
anti-mouse Neurofilament (Fisher Scientific MS359R7)
anti-mouse SSEA1 (Sigma MAB4301)
anti-mouse OCT4 (Santa Cruz sc-8628)
anti-mouse Smooth Muscle Actin (Abcam ab5694)
anti-mouse FOXA2 (Abcam ab108422)
anti-mouse SOX17 (R&D AF1924)
anti-mouse Troponin (cTNT, abcam, ab8295)
anti-mouse H-2Kb (Biolegend 116511)
anti-mouse H-2Kq (Biolegend 115106)

Anti-human
anti-human PDL1 (APC, Biolegend 329708)
anti-human FALSG (PE, Biolegend 306406)
anti-human CD200 (APC/Cy7, Biolegend 329220)
anti-human CD47 (PerCP/Cy5, Biolegend 323109)
anti-human SERPINB9 (ThermoFisher MA5-17648)
anti-human CCL21 (R&D AF366-SP)
anti-human MFGE8 (R&D IC27671A)
anti-human CD45 (APC, BD Biosciences 560973)
anti-human CD3 (Pe-Cy5, BD Biosciences 561006)
anti-human HLA-A,B,C (APC, Biolegend 311409)
anti-human CD31 (Pe-Cy7, Biolegend 303118)
anti-human CD144 (PE, Biolegend 138009)
anti-human CD16/CD32 Fc blocker (BD Biosciences 553141)
anti-human Human TruStain FxC Fc blocker (Biolegend 422301)

Secondaries and Others
anti mouse IgG (alexa488, ThermoFisher A10680)
anti goat IgG (alexa488 ThermoFisher A27012)
anti-Rat Fc (alexa568 secondary antibody (ThermoFisher A21112).
anti-hamster (Dylight 488 Biolegend 405503)
anti-rat IgG (DyLight 549 Novus NBP1-72975)
anti-mouse (alexa647 ThermoFisher 21244)
Annexin V (BV421, Biolegend 640923)
PI (Biolegend, 421301)

Validation

Antibody performance and validations are available from the vendor and/or manufacturer.

Experiments using antibodies included relevant negative biological controls, such as cells without transgene expression or undifferentiated cells. Antibodies relying on secondary antibodies also included secondary only-controls (no primary antibody).

Eukaryotic cell lines

Policy information about cell lines and Sex and Gender in Research

Cell line source(s)	Mouse C67BL6/N C2 embryonic stem cells were derived in the Nagy lab and in The Centre for Phenogenomics.<br>Human H1 embryonic stem cells were imported from WiCell.
Authentication	The cell lines were not authenticated before use.
Mycoplasma contamination	All cell lines and cultures were routinely tested for mycoplasma, and only negative cultures were used for experiments.
Commonly misidentified lines<br>(See ICLAC register)	No commonly misidentified cell lines were used.

Animals and other research organisms

Policy information about studies involving animals; ARRIVE guidelines recommended for reporting animal research, and Sex and Gender in Research

Laboratory animals	C57BL/6N (Strain 005304), C3H/HeJ (Strain 000659), FVB/NJ (Strain 001800), BALB/cJ (Strain 000651), and NSG mice (Stock 005557) were purchased from the Jackson Laboratory.<br>CD1 (Stock 022) mice were purchased from Charles River.
Wild animals	The study did not involve wild animals.
Reporting on sex	Both male and female recipients were used in the study.
Field-collected samples	The study did not involve samples collected from the field.
Ethics oversight	All mouse housing, husbandy, and procedures were performed in compliance with the Animals for Research Act of Ontario and the Guidelines of the Canadian Council on Animal Care. The animal protocols used in this study were approved by the Toronto Center for Phenogenomics (TCP) Animal Care Committee.

Note that full information on the approval of the study protocol must also be provided in the manuscript.

Flow Cytometry

Plots

Confirm that:

- [x] The axis labels state the marker and fluorochrome used (e.g. CD4-FITC).
- [x] The axis scales are clearly visible. Include numbers along axes only for bottom left plot of group (a 'group' is an analysis of identical markers).
- [x] All plots are contour plots with outliers or pseudocolor plots.
- [ ] A numerical value for number of cells or percentage (with statistics) is provided.

Methodology

Sample preparation	To generate Klg-1, Klg-2, and cloaked human lines, transfected mouse or human ESCs were collected at 60–80% confluency, washed with FACS buffer (PBS + 1% BSA and 0.5% EDTA), stained with antibodies, resuspended in FACS buffer + 25mM HEPES, filtered into Falcon tubes with cell-strainer lids, and then FACS-sorted on a MoFlow Astrios EQ sorter.<br><br>Cells collected for Flow cytometry analysis only (no sorting) were prepared in an identical fashion, except the final suspension buffer did not contain 25mM HEPES. Cells were run on a GALLIOS flow cytometer.
Instrument	For cell-sorting experiments (FACS), a MoFlo Astrios EQ cell sorter (Beckman Coulter, Miami, FL, USA) was used, equipped with 305-nm, 405-nm, 488-nm, 561-nm and 640-nm lasers.<br><br>For analysis, a GALLIOS flow cytometer, Beckman Coulter, equipped with 405-nm, 488-nm, 561-nm and 640-nm lasers.
Software	FACS software: Summit v6.2 (Beckman Coulter);<br>Analysis software (for Gallios data): Kaluza v1.5 (Beckman Coulter) and FlowJo V10
Cell population abundance	All lines that were FACs-sorted were analysed by post-sort analysis and compared to controls to ensure that the desired population was sorted. Extended Data Fig. 5b shows representative gating and cell-abundance graphs for the desired cell population.
Gating strategy	All samples were first gated for live single cells using side-scatter pulse height and area along with a viability dye. Cells

Gating strategy

without marker or factor expression, as well as single-color samples of the same cell type as the experimental samples, were used for negative controls to set gating and instrument compensation.

☒ Tick this box to confirm that a figure exemplifying the gating strategy is provided in the Supplementary Information.
